# Targeting of Embryonic Stem Cells by Peptide-Conjugated Quantum Dots

**DOI:** 10.1371/journal.pone.0012075

**Published:** 2010-08-10

**Authors:** Shuai Lu, Xing Xu, Wenxiu Zhao, Weiwei Wu, Hang Yuan, Huaibin Shen, Changhua Zhou, Lin Song Li, Lan Ma

**Affiliations:** 1 Department of Biological Sciences and Biotechnology, Tsinghua University, Beijing, China; 2 Life Science Division, Graduate School at Shenzhen, Tsinghua University, Shenzhen, China; 3 Kunming Yunda Monoclonal Antibody Technology Center, Yunnan University, Kunming, China; 4 State Key Laboratory for Supramolecular Structures and Materials, College of Chemistry, Jilin University, Changchun, China; 5 Key Laboratory for Special Functional Materials of the Ministry of Education, Henan University, Kaifeng, China; University of Southampton, United Kingdom

## Abstract

**Background:**

Targeting stem cells holds great potential for studying the embryonic stem cell and development of stem cell-based regenerative medicine. Previous studies demonstrated that nanoparticles can serve as a robust platform for gene delivery, non-invasive cell imaging, and manipulation of stem cell differentiation. However specific targeting of embryonic stem cells by peptide-linked nanoparticles has not been reported.

**Methodology/Principal Findings:**

Here, we developed a method for screening peptides that specifically recognize rhesus macaque embryonic stem cells by phage display and used the peptides to facilitate quantum dot targeting of embryonic stem cells. Through a phage display screen, we found phages that displayed an APWHLSSQYSRT peptide showed high affinity and specificity to undifferentiated primate embryonic stem cells in an enzyme-linked immunoabsorbent assay. These results were subsequently confirmed by immunofluoresence microscopy. Additionally, this binding could be completed by the chemically synthesized APWHLSSQYSRT peptide, indicating that the binding capability was specific and conferred by the peptide sequence. Through the ligation of the peptide to CdSe-ZnS core-shell nanocrystals, we were able to, for the first time, target embryonic stem cells through peptide-conjugated quantum dots.

**Conclusions/Significance:**

These data demonstrate that our established method of screening for embryonic stem cell specific binding peptides by phage display is feasible. Moreover, the peptide-conjugated quantum dots may be applicable for embryonic stem cell study and utilization.

## Introduction

Embryonic stem (ES) cells hold great promise for replacement therapies and studies of developmental biology, due to their ability to differentiate to all lineages of cells while maintaining an undifferentiated state during *in vitro* culture [1], [2], [3]. In ES cell studies, it is critical to find specific markers for ES cells. Several common makers for embryonic stem cells, such as Oct-4 and SSEAs, have been reported; however, some of these markers are not common for all ES cell lines [4], [5], [6]. Discovery of new ES cell markers is critical not only for identification, isolation, and visualization of ES cells, but also for potential clinical treatment as a targeting agent.

Phage display is one of the most efficient methods to identify novel biomarkers. The principle of the technology is based on fusing nucleotide sequences of random polypeptides to that of a phage coat protein which enables display of the chimeric proteins on the phage surface. This method provides a direct link between the selectable phenotype and reproducible genotype encoding for that phenotype. By selection with the target of interest, a phage pool that has increasing specific binding ability to the target can be obtained efficiently. Phage display serves as a valuable tool for selection of the new biomarkers for wide range of biomaterials [7], [8], [9], [10]. Among all biomaterials targeted for phage display selection, directly using whole cells as selection target has many advantages: 1) it needs no further information about cell surface molecules or purification of these molecules; 2) the surface molecules are most likely in their original state; 3) the selection stimulates the native binding environment where other macromolecules coexist. In this way, antibodies and peptides could be directly and quickly generated from cell screening, which are promising in the applications of cell labeling, gene delivery and ligand stimulation [11]. So far, various novel peptides and antibodies have been found using strategies of biopanning cells *in vitro* [12], [13], [14], [15].

Nanoparticles are a promising platform for stem cell study and manipulation [16]. The advantages of nanoparticles include large absorption cross section, slow photo-bleaching and low cytotoxicity [17]. Recently, several groups have reported that nanoparticles can be applicable to gene delivery, none-invasive imaging, and differentiation manipulation of embryonic stem cells [18], [19], [20], [21], [22], [23], [24], [25], [26]. Additionally, peptide-conjugated quantum dots can be successfully used as for labeling and targeting materials [27], [28]. However, the selectivity of peptide and subsequent specificity of nanoparticle targeting are still under investigation.

Here, we describe a method to screen for peptides that specifically bind to ES cells by phage display. By using this method, we found phages that display the APWHLSSQYSRT peptide demonstrated a high affinity and specificity to the undifferentiated embryonic stem cells in an enzyme-linked immunosorbent assay (ELISA). To exclude non-specific binding, we used the differentiated ES (dES) cells and primary mouse embryonic fibroblast (PMEF) cells, a feeder layer for the maintenance pluripotency of embryonic stem cells, as a control. Specific binding was further confirmed by immunofluoresence microscopy. To investigate whether this binding was caused by the displayed peptide rather than phage proteins, we chemically synthesized a peptide bearing an identical sequence. The competition experiment showed that the binding capability was conferred by the peptide sequence. We subsequently ligated the peptide to CdSe-ZnS core-shell nanocrystals. We found these peptide-conjugated quantum dots were able to specifically target the embryonic stem cells. We further showed that the binding of the selected peptide to undifferentiated embryonic stem cells was through a protein-protein interaction and that the tertiary structure of the peptide determined by molecular dynamic stimulation might provide an explanation for the interaction.

## Materials and Methods

### Cell preparations

Rhesus monkey embryonic stem cell line RS366.4 (kindly provided by Dr. James A. Thomson, The Wisconsin Regional Primate Research Center, University of Wisconsin, US) and mouse embryonic stem cell line M9 (kindly provided by Joint Lab of Stem Cell Research, Graduate school at Shenzhen, Tsinghua University, China) were plated on mitomycin treated PMEF cells, and cultured in ES medium comprised of 85% DMEM (Hyclone, Logan, UT), 1% nonessential amino acid (Gibco BRL, USA), 1 mM L-glutamine (Gibco BRL), 0.1 mM β-mercaptoethanol (Amresco, Solon, Ohio), and 15% fetal bovine serum (FBS) (Gibco BRL) as previously described [29]. For the characterization the undifferentiated state of ES cells, alkaline phosphatase assay, RT-PCR and immunofluorescence assay were performed. Alkaline phosphatase assay of ES cells was performed following the protocol as previous described [29]. For the RT-PCR assay, total RNA from ES cells was extracted by Trizol (Gibco BRL), reverse transcripted into cDNA by One-Step RNA PCR Kit (Takara) and PCR amplified using the primers and conditions in Table S2. Before selection by phages, ES cells were quickly detached by dispase (Gibco BRL) (5mg/ml), washed twice with PBS, and suspended in blocking buffer (PBS, 3% FBS) at 3×10^6^ cells/ml. For the preparation of differentiated ES (dES) cells, ES cells were harvested by digestion with dispase (5mg/ml). Detached ES cell colonies were plated in 0.1% gelatin (Sigma) pre-coated T75 flasks (Falcon, Becton Dickinson Labware, Franklin Lakes, NJ) and were subsequently grown in differentiation medium which contains 90% DMEM (Hyclone, Logan, UT) and 10% newborn calf serum (NCS) (Gibco BRL). After culture for two to three weeks, the ES cells underwent spontaneous differentiation. The dES cells were propagated and passaged by incubation with 0.05% trypsin/0.53 mM EDTA. PMEFs were cultured and passaged as previously described [29]. For preparation of phage display, dES cells and PMEF cells were quickly digested with 0.05% trypsin/0.53 mM EDTA, washed twice in PBS, and resuspended in blocking buffer at 1×10^6^ cells/ml for dES cells and 5×10^5^ cells/ml for PMEFs.

### Phage peptide library selection

The Ph.D-12 peptide phage display library was obtained from New England Biolabs (Beijing, China). M13 Phages from the Ph.D-12 library display 12 random amino acids at the N-terminus of the pIII coat protein. Initially, approximately 1.5×10^11^ phages were added to 1 ml of dES cell suspension, the mixture was subsequently incubated at room temperature for 1 hr with slow shaking, followed by centrifugation at 3000 rpm for 3 min. The supernatant that contains phages not binding to dES cells underwent two more subtractions with dES cells, followed by three subtractions with PMEFs using the same protocol. 1 µl of supernatant with subtracted phages were titered to determine the efficiency of subtraction. The remaining phages were mixed with 1 ml ES cell suspension, gently shaked at room temperature for 90 min, washed 5 times with washing buffer (PBS, 3% BSA, 0.1% Tween-20). For elution of specific binding phage, 1.6 ml elution buffer (0.1 M glycine-HCl, 0.1% BSA, pH 2.2) was added for no more than 10 min, and then was neutralized with 0.3 ml 1 M Tris-HCl, pH 9.1. The eluted phages were titered and amplified according to the manufacturer's protocol. In total, four round biopannings were performed in the same condition except that the concentration of Tween-20 in elution buffer was gradually increased from 0.1% to 0.5%. The binding efficiency of the phage pool was calculated by the ratio of its output phage number to input phage number in each round selection, in order to survey the effect of selection.

### Sequencing of phage DNA

The selected phages described above were used to infect *E.coli* strain ER2738, then the infected cells were plated, and monoclonal phages were rescued from phage plaques; the DNA from the amplified monoclonal phages were extracted and sequenced following the manufacturer's protocol. The corresponding peptide sequences were analyzed for similarity between themselves using Vector NTI software (Invitrogen).

### Whole-cell ELISA

The binding ability and specificity of the selected phages to ES, dES and PMEF cells were measured by enzyme linked immunosorbent assay (ELISA). Confluent cells in 96-well plate were fixed by 4% paraformaldehyde for 15 min, washed three times with PBS, blocked with 250 µl blocking buffer (PBS, 3% BSA), incubated at 37°C for 2 hrs, and washed three times with PBS. Subsequently, 2×10^9^ phages in blocking buffer were added and incubated for 90 min at room temperature. Then the plate was washed three times with PBS, 0.1% Tween-20, another three times with PBS, followed by incubation with HRP-conjugated mouse anti-M13 antibody (1∶1000 in blocking buffer) for 90 min at room temperature. After three washes with PBS, 100 µl o-phenylenediamine (OPD) substrate solution was added, and incubated for 30 min in dark, before being quenched by 50 µl 2 M H_2_SO_4_. The absorbance was measured at 492 nm by a GENios microplate reader (Tecan), and the absorbance at 405nm was taken as a reference.

### Determination of binding specificity by competition with synthesized oligopeptides

To analyze the binding ability of the selected phage to ES cells, chemically synthetic peptides were added at gradient concentrations from 10 nM to 10 µM to cells before addition of phages during the process of biopanning. A randomly scrambled peptide was used as a negative control. The binding ratio of phages to ES cells was determined by the ratio of output titer of phages selected by adding peptide normalized to the output titer of phages selected without adding peptides. Each experiment was repeated three times independently.

### Immunofluorescence microscopy and internalization assay

To further confirm the result of Whole-cell ELISA, immuncytochemistry was carried out. ES, dES and PMEF cells plated in 24-well plate (Falcon, Becton Dickinson Labware, Franklin Lakes, NJ) were washed twice with PBS, fixed with 4% paraformaldehyde for 15 min at room temperature. After three washes with PBS, 2×10^9^ phages in 200 µl PBS, 1% BSA were added into each well. After incubation for 90 min at 37°C or 25°C, the plate was washed three times with PBS, followed by the addition of Mouse anti-M13 antibody (1∶1000 diluted in PBS, 1% BSA) for 1 hr at 37°C. The plate was then washed three times with PBS, and incubated with FITC-conjugated goat anti-mouse IgG (1∶100 diluted in PBS, 1% BSA) (Sigma-Aldrich) for 1 hr at 37°C in dark. Cells were counterstained with Hoechest 33258 (Invitrogen) for 15 min and photographed by an Eclipse TE2000 fluorescence microscopy (Nikon). The photographs were processed with Image-Pro Plus software (Media Cybernetics, Inc). The assay was performed independently for three times. For the internalization assay, cells were first incubated with 2×10^9^ phages in PBS/1% BSA, and then washed with wash buffer (100 mM glycine, 0.5 M NaCl, 1% BSA, pH 2.5) three times to wash away non-internalized phages, and permeated by 0.1% Triton X-100 before paraformaldehyde fixation.

### Western blotting

Total proteins from Rhesus ES cells were extracted by 20 mM Tris, 200 mM NaCl, 1 mM EDTA, 0.5% NP-40, 1% SDS, protease inhibitor cocktail (Roche, Nutley, NJ), and resolved on 12% SDS-PAGE gel, followed by electrophoretical transferring to PVDF membrane (Millipore, Billerica, MA). The blot was blocked with PBS, 5% BSA for 1 hr, and subsequently incubated with 2×10^8^ phages in PBS, 3% BSA for another hour. After three washes with PBS, the membrane was incubated with HRP-conjugate mouse anti-M13 antibody (1∶1000 dilution in PBS, 5% BSA) for 1 hr. The membrane was further developed with ECL western blotting substrate (Pierce, Rockford, IL), and the signal was detected by an Omega 12ic molecular imaging system (Ultra-LUM).

### Quantum dot peptide conjugation and labeling cells

CdSe-ZnS core-shell quantum dots (QDs) were prepared according to the previously published method [29]. The QDs with emission maxima centered at 580 nm were conjugated with peptides via covalent bonding using EDC. The peptides were reacted with Sulfo-NHS bound QDs for 2 hr at room temperature and then stored at 4°C. QD-labeled peptides were incubated with cells to identify a specific binding ability of selected sequence. Cells were plated in 1% gelatin coated 24-well plate. After adherence, the cells were washed twice with PBS, mixed with 5 µL QD-labeled peptide and 100 µL 4% paraformaldehyde in 400 µL blocking buffer with gentle shaking at room temperature for 1 hr, followed by washing three times with 0.1% Tween-20-PBS. Cells were counterstained with Hoechest 33258 (Invitrogen) for 15 min at room temperature and detected by FV1000 Confocal laser scanning biological microscope (Olympus). Free quantum dots without peptide conjugation were set as a negative control.

### Molecular dynamics stimulation of peptide structure

Initially, an unfolded and fully extended structure was generated by PROTEIN program in Tinker software (http://dasher.wustl.edu/tinker/) [30] (backbone torsion ϕ, ψ = −135, 135), using OPLS-AA force field parameters to assign the atom types. The structure was subsequently optimized by Truncated Newton Conjugate Gradient method using GB/SA continuum solvation model. Afterward 1 ns molecular dynamics stimulation was carried out at a time step of 2.0 fs, and the system thermostat temperature was targeted to 298 K. The result of molecular dynamics stimulation was further rendered with POV-RAY program.

## Results

### Screen of specific ES cell binding peptides by phage display

**Figure 1:**
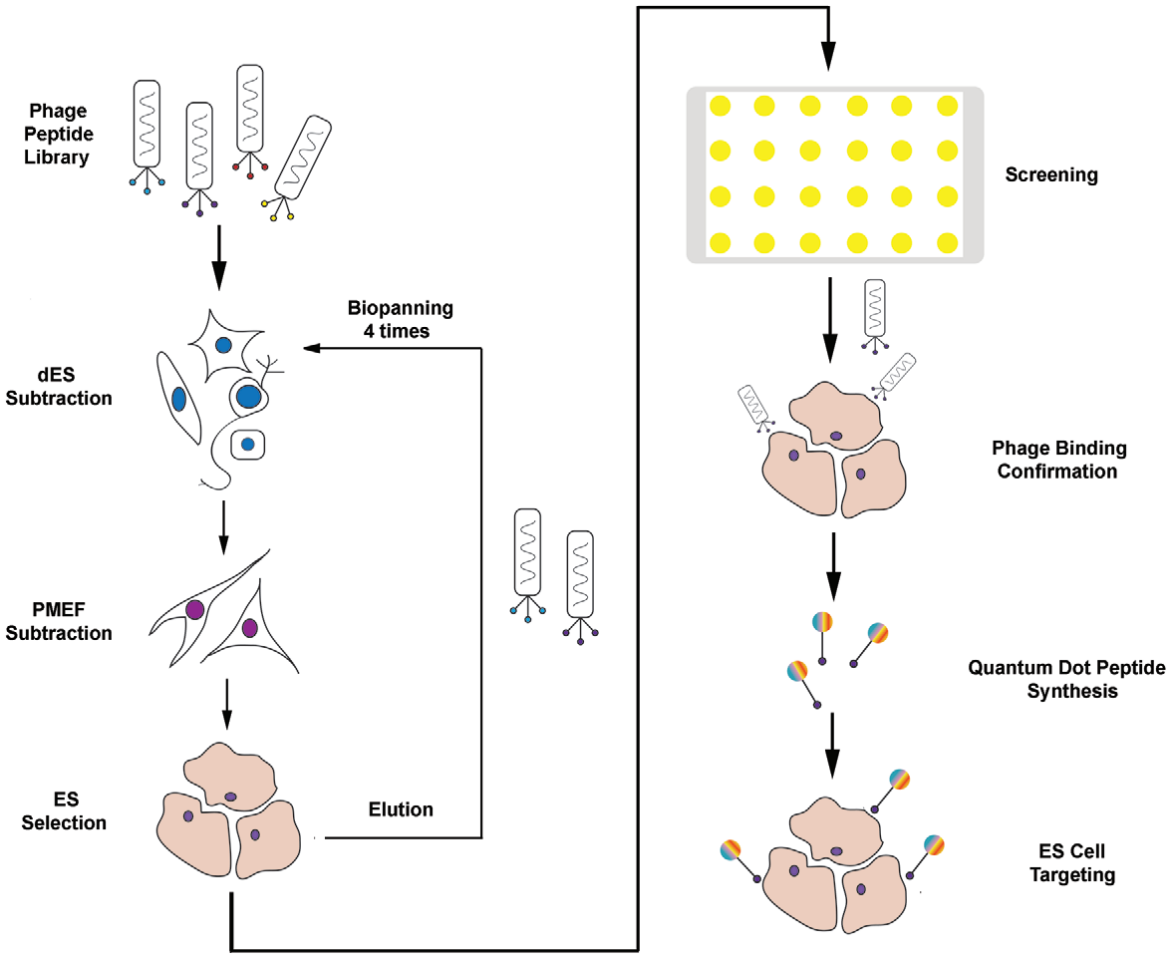
Scheme of experiment design. Initially, phages that specifically bind to ES cells were enriched in phage pool by four rounds of biopanning. Each round of biopanning included three subtraction steps with dES cells, three subtraction steps with PMEFs and a selection step with ES cells. After enrichment, monoclonal phages were purified and screened by enzyme linked immunosorbent assay (ELISA). Phages that showed higher binding ability and specificity were further confirmed with immunofluorescence microscopy. Then the selected peptide was chemically linked with quantum dots and the subsequent peptide-quantum dot conjugates were tested for ES cell targeting.

**Figure 2:**
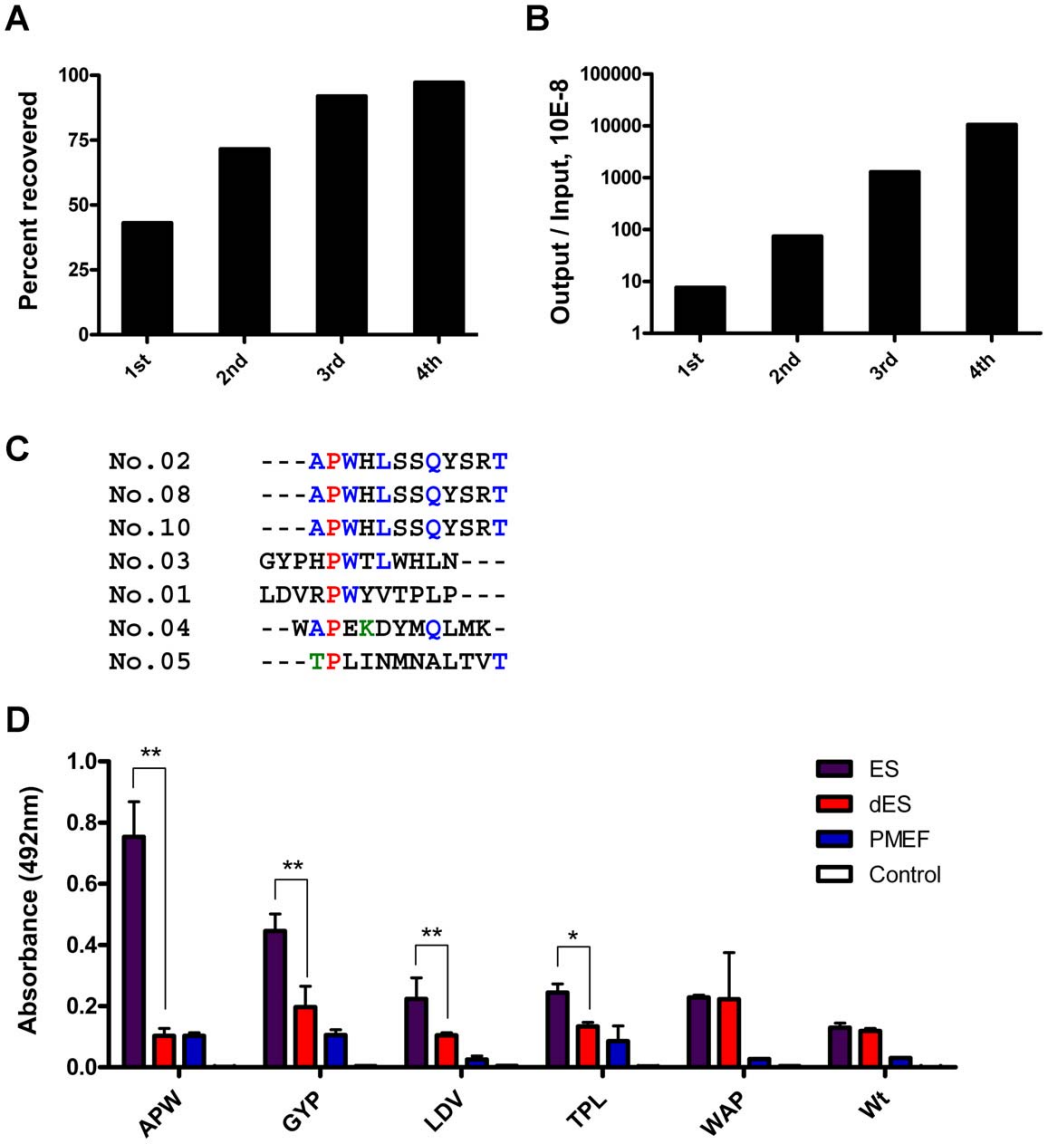
Selection of Ph.D-12 phage pools against ES cells. (A) Subtraction efficiency with dES cells and PMEFs. The phage pool underwent three subtractions with dES cells and three subtractions with PMEFs in each round of biopanning. The efficiency is shown as the percentage of recovered phage numbers from subtraction to input phage numbers. (B) Selection efficiency with ES cells. The binding efficiency of phage pool is shown as the percentage of eluted phage numbers to input phage numbers in each round of biopanning. (C) Alignment of peptide sequences displayed by monoclonal M13 phages selected binding to ES cells. Identical sequences are shown in color. (D) Affinity and specificity of purified candidate M13 phages binding to indicated cell types using ELISA. The peptides displayed by selected phages are APWHLSSQYSRT, GYPHPWTLWHLN, LDVRPWYVTPLP, TPLINMNALTVT, and WAPEKDYMQLMK. The first three amino acid letters of these sequences are used to stand for the corresponding phages. Results are presented as absorption value (mean ± standard derivation) in ELISA assay. Each experiment was repeated three times independently. **: p<0.01, and *: p<0.05 using ANOVA test. BSA and M13 wild type phage (Wt) were used as the negative controls.

Phages that specifically bound to ES cells were enriched in the phage pool by four rounds of biopannings. Each round of biopanning included three subtraction steps with dES cells, three subtractions steps with PMEFs and then a selection step with ES cells (Figure 1). During four rounds of biopannings, phage pools exhibited decreasing binding efficiency to differentiated ES (dES) cells and PMEFs. We observed the increasing recovery rate after subtraction, from 43% up to 97%, indicating that the subtraction step in our strategy was efficient to reduce non-specific binding of phages to ES cells (Figure 2A). Selected phage pools also showed increased binding efficiency to ES cells, from 8×10^−8^ to 1×10^−4^, indicating successful accumulation of specific ES cell binding phages (Figure 2B).

After four rounds of biopannings, monoclonal phages were randomly picked from the selected phage pool and the genomes of the phages were sequenced. Of ten phage clones that had been picked, three displayed the same peptide sequence APWHLSSQYSRT and four displayed peptides showed homologous sequence with APWHLSSQYSRT (Figure 2C). The sequences of the peptide region of the remaining three phages showed deletion (data not shown). We noticed that a PWX (L/V) motif (Figure 2C) co-exists in the APW, GYP and LDV phages. This suggests that the specific binding ability might be due to the PWX (L/V) motif. However specificity it is not determined solely by the consensus motif but is also through the coordination by other flanking amino acids since the binding affinities of these phages are not identical.

Binding affinities and specificities of the purified phage candidates were further analyzed by whole-cell ELISA. Of all candidates the APW phage (APWHLSSQYSRT), GYP phage (GYPHPWTLWHLN) and LDV phage (LDVRPWYVTPLP) showed significantly greater binding efficiency to ES cells than to dES cells, PMEFs and the negative control (p<0.01); the TPL phage (TPLINMNALTVT) showed significant greater binding efficiency as well (p<0.05), while the WAP phage (WAPEKDYMQLMK) showed no significantly specific binding (Figure 2D). Since the APW phage showed the greates binding specificity to ES cells and was most enriched in the phage pool, we decided to further investigate and confirm its binding with ES cells.

### Confirmation of the binding capability and specificity of the selected phage to ES cells

**Figure 3:**
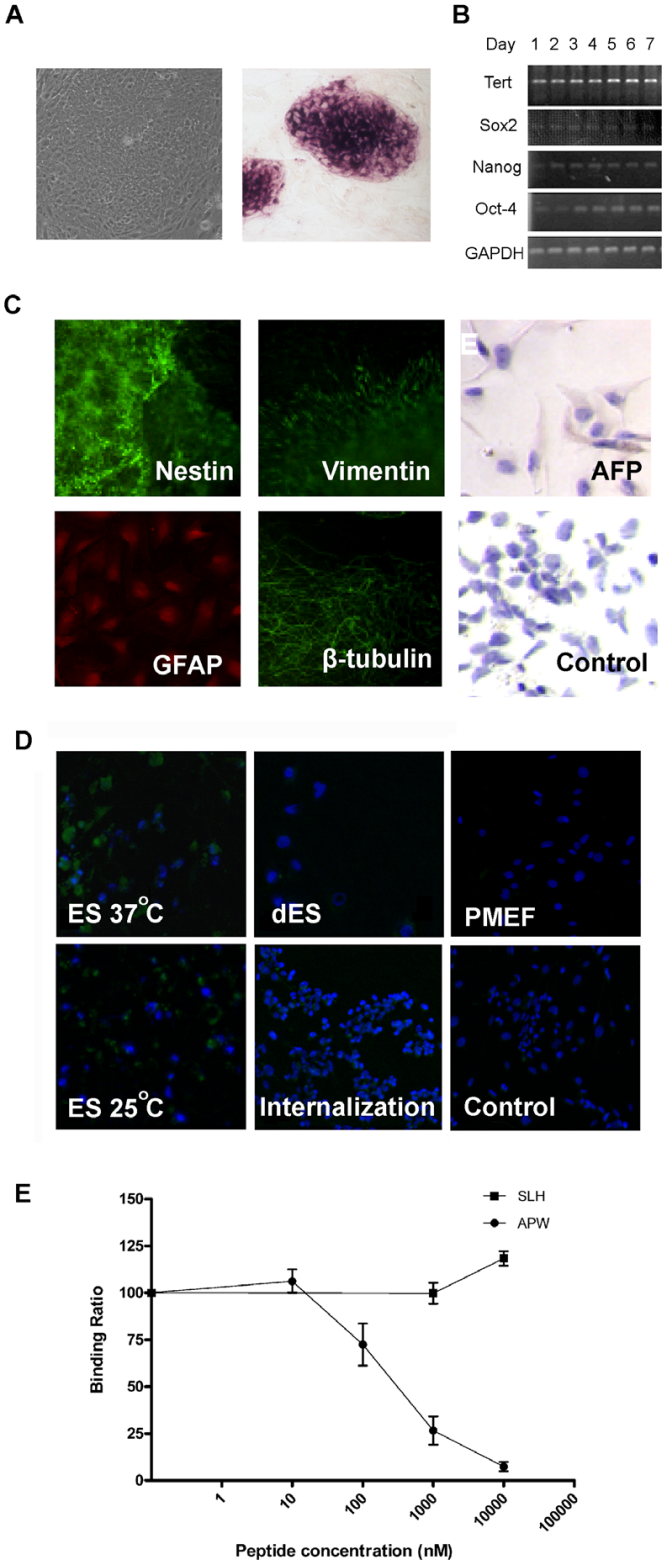
Confirmation of binding affinity and specificity of selected phage to ES Cells. (A) Bright-field image of an ES cell colony and alkaline phosphotase characterization of ES cell colonies. (B) RT-PCR detection of ES cell marker gene expression. (C) Immunofluorescence and immunochemistry detection of differentiated ES cell markers. (D) Immunofluorescence microscopy of APW phage binding to ES cells, dES cells and PMEFs. APW phage binding ability to ES cells was tested at both 37°C and 25°C. Binding ability of APW phage to dES cells and PMEFs as well as internalization of APW phage by ES cells were tested at 37°C. Wildtype M13 phage was used as a control. The immunofluorescence signal of M13 phage is shown in green. Cell nuclei were counter-stained with Hoechest 33258 (blue). (E) Competition of APW phage binding by chemically synthesized peptides, APWHLSSQYSRT and SLHSQPRSWTAY. Increasing concentrations of the peptide from 10 nM to 10 µM were added to 2×10^6^ ES cells before addition of 1×10^10^ APW phages. Binding ratio was determined by output titer of phages with addition of peptides normalized to that without addition of peptides. Each experiment was repeated three times and the values were shown as mean ± standard derivation.

In order to confirm our ES cell line is maintaining the undifferentiated state, we first checked the undifferentiated state makers in our ES cell line. The ES cells formed round, compact and clearly defined colonies as previously described [29] (Figure 3A, left). Also, ES cell colonies were positive in the alkaline phosphatase assay, another characteristic of undifferentiated ES cells (Figure 3A, right). We then monitored the expression levels of ES cell marker genes by RT-PCR. The mRNAs of Octamer binding protein 4 (Oct-4), telomerase reverse transcriptase (Tert), Nanog, and SRY (sex determining region Y)-box 2 (Sox2) are expressed in the ES cell lines, further confirming the undifferentiated state of ES cells (Figure 3B). In order to determine whether ES cells are able to differentiate to cells from all three germ layers, we tested the marker protein expression in dES cells. The result showed that the dES cells were positively stained with Nestin (ectoderm), Glial fibrillary acidic protein (GFAP) (ectoderm), Vimentin (mesoderm), β-tubulin (mesoderm) and alpha-fetoprotein (AFP) (endoderm), indicating that the ES cells can be differentiated to all three germ layers (Figure 3C). Additionally, the ES cells could form the embryonic bodies in suspension culture system, showing their pluripotency (Figure S1).

Immunofluorescence microscopy was performed to confirm that the APW phage has the specific binding ability to ES cells. APW phages were able to bind to ES cells (green signal in Figure 3D). No obvious fluorescent signal of dES cells and PMEF cells was observed. Wild type M13 phage did not bind to ES cells, as shown in the control. We also carried out the internalization assay for APW phages, which showed a weak signal (Figure 3D). GYP phage and LDV phage also showed specific binding to ES cells; however, their affinities were much lower when compared to the APW phages (data not shown). We subsequently tested whether a chemically synthesized peptide bearing the APWHLSSQYSRT sequence could compete with the APW phage for the binding to ES cells. As shown in Figure 3E, the chemically synthesized APWHLSSQYSRT peptide was able to compete with the APW phage, starting at the concentration of 100 nM and achieving 50% inhibition at the concentration of about 500 nM. An SLHSQPRSWTAY peptide with randomly scrambled sequence of the APWHLSSQYSRT peptide failed to compete with the APW phage for the binding to ES cells. These data demonstrate that the binding ability of the phage to ES cells is caused by it displaying the APWHLSSQYSRT peptide.

### Targeting of ES cells by the peptide-quantuam dot conjugates

**Figure 4:**
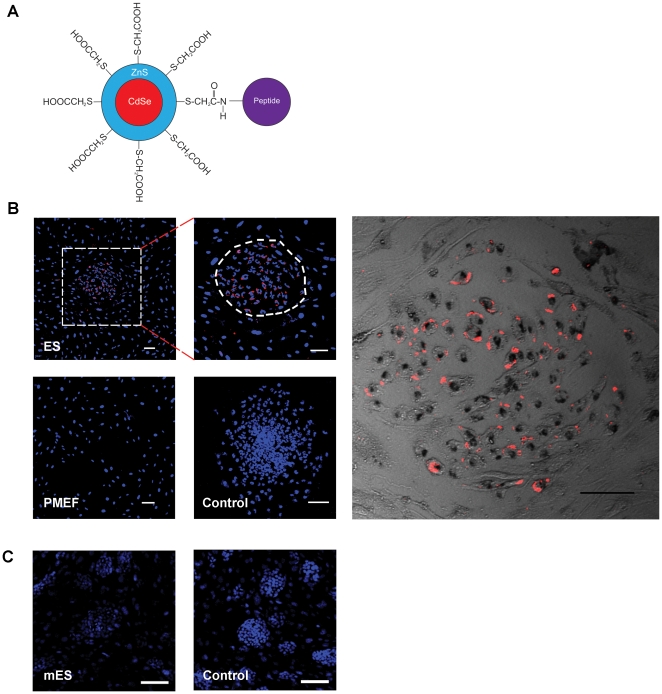
Targeting of ES cells by peptide-conjugated quantum dots. (A) Scheme of the peptide-conjugated quantum dots. (B) Bright-field and fluorescence microscopy of APWHLSSQYSRT peptide-conjugated quantum dots targeting ES cells. Dashed circle shows an ES cell colony. The fluorescence signal of peptide-conjugated quantum dots is shown in red and cell nuclei are shown in blue (Hoechest 33258). PMEF cells and quantum dots without conjugation of peptides were set as the controls. Scale bar, 100 µm. (C) Fluorescence microscopy of APWHLSSQYSRT peptide-conjugated quantum dots with mouse ES (mES) cells. The binding of APWHLSSQYSRT peptide-conjugated quantum dots to mES cells were undetectable. Free quantum dots without conjugation of peptides were set as the control. Scale bar, 100 µm.

CdSe-ZnS quantum dots were conjugated with the APWHLSSQYSRT peptide via stable covalent bonding using EDC, a crosslinking agent that couples carboxyl groups to the amino groups of the peptide (Figure 4A). We then investigated whether the peptide conjugated quantum dots were able to target ES cells. By incubating the APWHLSSQYSRT peptide-quantum dots with ES cells, followed by confocal laser scanning microscopy, we found that the conjugates can bind to ES cells efficiently. As shown in Figure 4B, the conjugates specifically target to the ES cell colonies (inside dashed line) rather than the PMEF cells (outside dashed line), which are used as a feeder layer. We also observed that the peptide-quantum dot conjugates target the ES cell surface with preference to the periphery of the cell surface, suggesting that the binding is specific to a particular compartment of the ES cell surface (Figure 4B, Bright-field image). In order to further test the binding specificity of the APWHLSSQYSRT peptide-quantum dots to ES cell lines, we assay the binding of the APWHLSSQYSRT peptide-quantum dots to a mouse ES cell line. As shown in Figure 4C, the APWHLSSQYSRT peptide-quantum dots did not bind to the mouse ES cell line. Also, there is no non-specific binding of the quantum dots to the ES cells by using the peptide-free quantum dots as the control.

### Selected peptide binds to ES cells through the protein-protein interation

**Figure 5:**
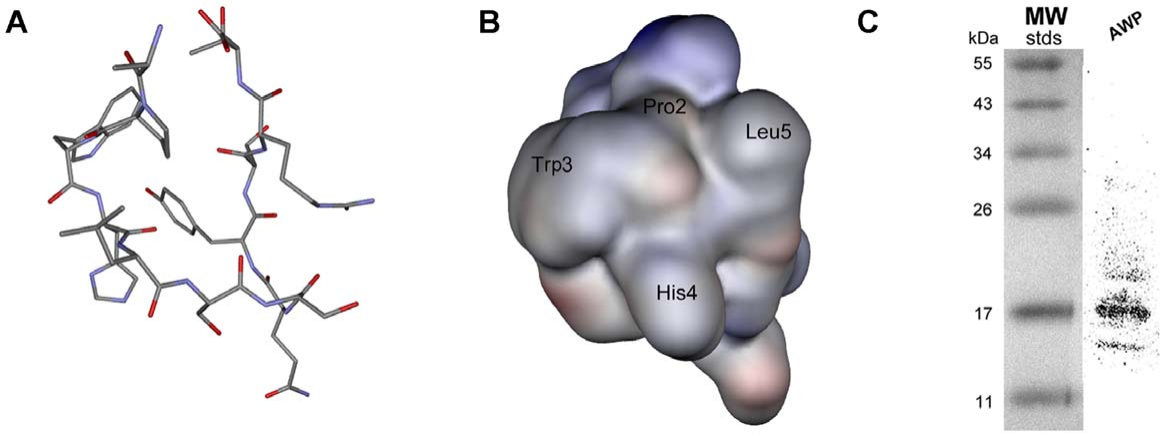
Molecular dynamics simulated structure of APWHLSSQYSRT peptide. (A) Stick model showing the scheme of folding with hydrogen hidden. (B) Surface model showing the possible binding site and selected side chains (Proline 2, Tryptophan 3, Histidine 4 and Leucine 5). (C) Western blotting result of APW phage binding to ES cell proteins. A major band was detected at 17 kDa and a minor band was detected at around 15 kDa.

To test whether the APW phage binds to ES cells through the protein-protein interaction, proteins extracted from ES cells were resolved on a 12% SDS-polyacrylamide gel and transferred to a PVDF membrane. After incubation with APW phages, a major band at 17 kDa and a minor band at about 15 kDa were observed (Figure 5C).

To better understand the molecular architecture and biological function of the APW peptide, we used the molecular dynamics stimulation to initially characterize and predict its structure. After 1 ns of folding simulation, the peptide was folded into a compact structure, with hydrophobic amino acid residuals facing one side while hydrophilic residuals facing another (Figure 5A). Furthermore, Trp, Pro and Leu residuals were relatively close in the spatial structure (Figure 5B). Combined with the fact that PWX (L/V) motif might be important for the peptide binding mentioned above, it is possible that the Trp, Pro and Leu residues form a hydrophobic environment which can be recognized by certain cell surface proteins.

## Discussion

In the present study, we have described a method to enrich phage pools that have specific binding efficiency and selectivity for ES cells. In order to eliminate the possibility of selecting non-relevant phages, we employed two steps of subtraction using dES cells and PMEFs before each selection step. The result showed that the subtraction is rather effective to exclude non-specific binding phages. In addition, the method that we chose must pass through a procedure of detaching cells from culture flasks by dispase or trypsin, which is thought to possibly change the composition and configuration of cell surface proteins [31]. To avoid the change of cell surface proteins, we shortened the digestion time as far as possible. We also tried the method using attached cells, however, they were apt to detach during the incubation and the concentration was much lower than that of suspended cells.

After the enrichment step, we picked several phages and sequenced their peptide display gene region. We found an APWHLSSQYSRT peptide sequence was the most accumulated during biopannings and several peptides contain a PWX (L/V) motif. The binding efficiency and specificity of the phage to ES cells was tested by whole-cell ELISA and the result showed that the APW phage served as a better candidate than other phages, which was further verified by the immunofluorescent analysis. We observed a phenomenon that these phages displayed a common motif but showed different binding ability further suggests that the motif is important for the binding while this binding ability is coordinated by flanking sequences. Furthermore, the result that the chemically synthesized APWHLSSQYSRT peptide could inhibit binding of the phage to ES cells indicated that the specific binding ability of APW phages was due to the displayed peptide and independent of the phage itself. A BLAST search of the sequence in the GeneBank showed several existing and/or predicted homologous sequences (Table S1). In these sequences, we find that several of them are of special interest. For example, human Chorionic Gonadotropin (hCG) that shares the homology with the APWHLSSQYSRT sequence is found to be important for development and leishmanolysin-like protein is reported to have the different expression pattern among different embryonic stem cell lines [32], [33]. These findings suggest the APWHLSSQYSRT peptide might simulate protein-protein interaction.

To further characterize the phage and the displayed peptide, we carried out western blotting. The result showed that the phage bound to ES cells by protein-protein interaction, which is consistent with the earlier report that peptides isolated from phage display often bind to the sites by protein-protein interaction [34], [35]. This finding also increases the possibility that the APW peptide has the ability to target cell surface proteins, which is possibly a membrane protein with a relatively low molecular weight (about 17 kDa). Further *in silico* studies of the peptide structure showed that the peptide contained a hydrophobic site and had a hydrophilic environment on the other side, which suggests that the peptide binds to ES cells via the non-bonding forces. Since generally proteins contain the core hydrophobic residues which are surrounded by a shell of hydrophilic residues, it is possible that the PWX (L/V) motif is important for the maintenance of tertiary structure of the peptide [36]. Combined with our previous experiments and the *in silico* studies, it is possible that the PWX (L/V) motif serves as an important core motif that binds to the ES cell membrane proteins. Additionally, the *in silico* studies of the peptide may open a perspective for improvement of its binding affinity and specificity by redesigning of the peptide. After conjugation with the peptide, the quantum dot showed a much higher binding ability to ES cells compared to the naked quantum dots, suggesting that adding the peptide can increase quantum dot binding affinity and specificity towards ES cells. Moreover, the fact that the peptide-conjugated quantum dots were unable to target mouse ES cell line further suggests their specificity towards a particular cell line.

In general, there are two major concerns in nanoparticles cell-targeting studies: specificity and toxicity. To increase the specificity of nanoparticles to cells, there could be targeting materials designed to link with the nanoparticles. Phage display is a useful tool for searching for the targeting materials. Our group and several other groups have successfully found peptides or antibodies that can specifically recognize a variety of stem cells using phage display [12], [13], [37], [38], [39]. However, it is still an open question that how specific these peptides or antibodies are. Although in our study, the selected peptide was unable to bind to the stem cell line of the other species, further proves its specificity, we still observed that several peptides with “homologous core sequence”, namely the PWX (L/V) motif, were able to bind to differentiated ES cells, albeit with low efficiency. Although this might be due to the incomplete differentiation of ES cells or the ES cells slightly differentiated during the selection step, so that some types of differentiated cells can not be distinguished from ES cells, it also possible that the recognition specificity to a particular cell is a relative value. Based on the result of our study, this value is possibly influenced by the flanking sequences of the core motif. By engineering the flanking sequences of the peptide, it is quite possible to increase the specificity of the peptide recognition and the *in silico* study might provide a possible direction for the engineering. Another concern of the nanoparticle cell-targeting study is the toxicity. Previous studies have shown that coating is the most important factor for cytotoxicity of the quantum dots [40], [41]. Another study has shown that the toxicity and metabolism of the quantum dots are different in different type of cells [42]. These studies suggest that the cytotoxicity of quantum dots can be minimized by proper coating technique and selection of correct type of quantum dot material according to the cell type of interest. However, the toxicity of the peptide-quantum dot conjugates remains to be investigated. Although previous studies have shown that targeting of CdSe-ZnS to other type of cells does not change their cellular morphology and physiology [28], [43], [44], the physiological effect of the peptide-quantum dot conjugates on ES cells is unknown.

In summary, we have established a method of screening for ES cell binding peptides by phage display and identified a novel peptide that specifically binds to ES cells. This peptide holds the promise to be a targeting or imaging agent for ES cells and might serve as a novel research tool for studying the ES cell surface as well. However, further studies are needed to understand the biology of the APWHLSSQYSRT peptide. Work is continuing to further assess its specificity using more cell types, to test whether this binding ability is the same *in vivo* as *in vitro*, to measure the toxicity of the peptide-quantum dot conjugates on ES cells and determine whether the peptide has a physiological effect on ES cells.

## Supporting Information

Figure S1 Embryonic body (EB) formation by differentiated embryonic stem cells. From day 1 to day 3, suspended cultured ES cells aggregate and form simple EB. After day 4, the EB form basal lamina (smoothen edge) and a central cavity occurs.
(1.86 MB TIF)

Table S1 Homological sequences identified by protein database search.
(0.03 MB DOC)

Table S2 Summary of RT-PCR primers for detection of undifferentiated ES cell marker gene expression.
(0.03 MB DOC)
