# Supplementary figures and images for: Targeting of Embryonic Stem Cells by Peptide-Conjugated Quantum Dots
Source: PLoS One. 2010 Aug 10;5(8):e12075. doi: 10.1371/journal.pone.0012075 (PMC2919412; doi:10.1371/journal.pone.0012075)

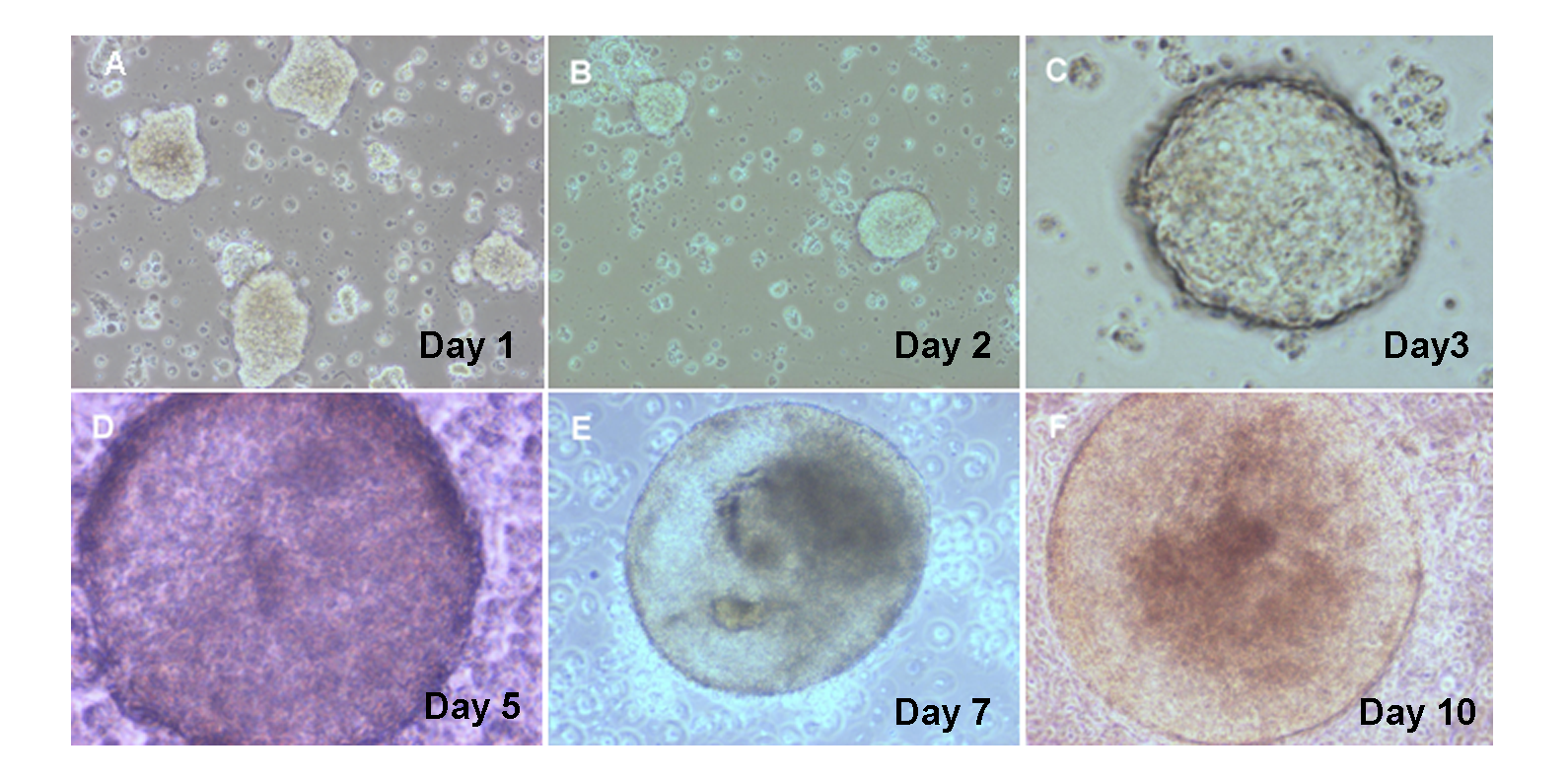

Supplement: Figure S1 — Embryonic body (EB) formation by differentiated embryonic stem cells. From day 1 to day 3, suspended cultured ES cells aggregate and form simple EB. After day 4, the EB form basal lamina (smoothen edge) and a central cavity occurs. (1.86 MB TIF) [file pone.0012075.s001.tif]
