# Supplementary material for: Targeting of Embryonic Stem Cells by Peptide-Conjugated Quantum Dots
Source: PLoS One. 2010 Aug 10;5(8):e12075. doi: 10.1371/journal.pone.0012075 (PMC2919412; doi:10.1371/journal.pone.0012075)
Supplement: Table S1 — Homological sequences identified by protein database search. (0.03 MB DOC) [file pone.0012075.s002.doc]

| AAB26811  XP_071712  EAX09028  XP_001082366  NP_998764  EAW90304  NP_006314  CAI12970  XP_001082366  XP_001084361  XP_001114526  XP_001091112 | peripheral myelin protein (*Homo sapiens*)  hypothetical protein (*Homo sapiens*)  hCG2045796 (*Homo sapiens*)  leishmanolysin-like (metallopeptidase M8 family) (*Homo sapiens*)  transmembrane protein 16E (*Homo sapiens*)  PITPNM family member 3 (*Homo sapiens*)  SEC24 (*S. cerevisiae*) homolog B (*Homo sapiens*)  pancreatic elastase IIB (*Homo sapiens*)  Predicted: peripheral myelin protein 22 isoform 1 (*Macaca mulatta*)  Predicted: hypothetical protein (*Macaca mulatta*)  Predicted: integrin alpha 2b (*Macaca mulatta*)  Predicted: disintegrin-like and metalloprotease (*Macaca mulatta*) | 1. WHLNSDYS 131   389 HLSSHAQYS 397  47 APWRQLSSQF 56  446 WHLSGEYQR 454  710 WKLTTQYRRT 719  16 APWHL 20  867 LSSQYS 872  25 PWQVSLQYS 33   1. WHLNSDYS 63   112 PWRLDSQY 119  245 WHVSSQ 250  971 WHFSEWSQCSR 981 |
| --- | --- | --- |

**Table S1.** Homological sequences identified by protein database search.
