# Supplementary material for: Targeting of Embryonic Stem Cells by Peptide-Conjugated Quantum Dots
Source: PLoS One. 2010 Aug 10;5(8):e12075. doi: 10.1371/journal.pone.0012075 (PMC2919412; doi:10.1371/journal.pone.0012075)
Supplement: Table S2 — Summary of RT-PCR primers for detection of undifferentiated ES cell marker gene expression. (0.03 MB DOC) [file pone.0012075.s003.doc]

| **Primer** | **Tm** | **Sequence** |
| --- | --- | --- |
| Tert | 53°C | F:5'-CCTGAAAGCCAAGAACGC-3'  R:5'-AGTAGGGGACGGACTAAACC-3' |
| Sox2 | 51°C | F:5'-CCCTGTGGTTACCTCTTCC-3'  R:5'-CTCCCATTTCCCTCGTTT-3' |
| Nanog | 56°C | F:5'-TTATTGTGGCGGTGACTC-3'  R:5'-TTGCCTTTGGGACTGGT-3' |
| Oct-4 | 54°C | F:5'-GACAACAATGAGAACCTTCA-3'  R:5'-CACATCCTTCTCTAGCCCAA-3' |
| GAPDH | 54°C | F:5'-TGAAGGTCGGAGTCAACGGA-3'  R:5'-TGGTGCAGGAGGCATTGCTG-3' |

**Table S2**. Summary of RT-PCR primers for detection of undifferentiated ES cell marker gene expression.
